# Functional connectivity of the cortical network supporting statistical learning in musicians and non-musicians: an MEG study

**DOI:** 10.1038/s41598-017-16592-y

**Published:** 2017-11-24

**Authors:** Evangelos Paraskevopoulos, Nikolas Chalas, Panagiotis Bamidis

**Affiliations:** 1 School of Medicine, Faculty of Health Sciences, Aristotle University of Thessaloniki, P.C., 54124 Thessaloniki, Greece; 2 Institute for Biomagnetism and Biosignalanalysis, University of Münster, P.C., D-48149 Münster, Germany; 3 School of Biology, Faculty of Science, Aristotle University of Thessaloniki, P.C., 54124 Thessaloniki, Greece

## Abstract

Statistical learning is a cognitive process of great importance for the detection and representation of environmental regularities. Complex cognitive processes such as statistical learning usually emerge as a result of the activation of widespread cortical areas functioning in dynamic networks. The present study investigated the cortical large-scale network supporting statistical learning of tone sequences in humans. The reorganization of this network related to musical expertise was assessed via a cross-sectional comparison of a group of musicians to a group of non-musicians. The cortical responses to a statistical learning paradigm incorporating an oddball approach were measured via Magnetoencephalographic (MEG) recordings. Large-scale connectivity of the cortical activity was calculated via a statistical comparison of the estimated transfer entropy in the sources’ activity. Results revealed the functional architecture of the network supporting the processing of statistical learning, highlighting the prominent role of informational processing pathways that bilaterally connect superior temporal and intraparietal sources with the left IFG. Musical expertise is related to extensive reorganization of this network, as the group of musicians showed a network comprising of more widespread and distributed cortical areas as well as enhanced global efficiency and increased contribution of additional temporal and frontal sources in the information processing pathway.

## Introduction

The human ability to extract regularities underlying the arrangement of the stimuli within a given stream, independently of the perceptual modality, is referred to as statistical learning. This process causes the segmentation of the stimulation stream into chunks according the transitional probabilities within the stimulus material^1,2^, and has been evidenced in both infants as well as adults^3,4^. This ability is of great importance for the detection and representation of regularities in the environment and therefore for its prediction, on the basis of implicit learning^5,6^. Recent neuroscientific studies indicate that statistical learning is a general mechanism that is applied in the processing of any type of sensory input and for a range of stimuli features; therefore it may be comprised by learning principles and neural underpinnings that ground on both, domain general and domain specific mechanisms^7^.

A vast variety of neuroscientific studies during the last decades indicate that brain function and structure can be substantially modified through intensive training^8^. Music training, especially, is known to reorganize perception and induce cortical plasticity related to the processing of auditory events^9^. Recent neuroimaging studies indicate that long term musical training may enhance implicit learning of auditory input and increase the ability to segment a series of tones or syllables on the basis of the underlying distributional properties of the corresponding stream^10^. While behaviorally, the result of this enhancement may not be evident^10,11^, neurophysiological evidence indicate an increase in the activity elicited by the auditory cortex related to unfamiliar items. This training effect has been documented by a previous magnetoencephalographic (MEG) study of our group^11^ that showed an increased positivity at the range of 50 ms after the onset of the unfamiliar sounds in the group of musicians. Nonetheless, in the same study, a statistical mismatch negativity response^6^ was generated after the onset of the unfamiliar sounds, which did not differ between musicians and non-musicians. Relevant electroencephalographic studies show a music training effect at the range of N1^12^ and N400^13^, while a music training effect in both behavioral and electrophysiological level is presented in the longitudinal study of Francois *et al*.^14^, highlighting the causal role of music training in the improvement of speech segmentation skills.

Several neuroimaging^15,16^ or lesion^17^ studies have identified cortical regions that may contribute to this process. These include temporal sources, inferior frontal gyrus (IFG), inferior parietal cortices, and the medial temporal lobe^11,15^. Nonetheless, complex cognitive processes such as statistical learning usually do not emerge as a result of activation in isolated brain regions, but rather from widespread cortical areas functioning in dynamic networks. Within this framework, Rodríguez-Fornells, *et al*.^18^, proposed that a complex network of brain substrates supports language learning, considering statistical learning as one of the main cognitive mechanisms underlying this process. Additionally, a recent study by De Diego Balaguer *et al*.^19^, revealed that the extraction of and learning of new rules, embedded in an artificial language, is related to increased gamma band phase coherence between frontal, temporal, and parietal regions, indicating a long range coherence of the corresponding cortical dynamics. In relation to statistical learning of tone sequences, a recent MEG study by Farthouat, *et al*.^20^, indicated that the left supplementary motor area and left posterior superior temporal sulcus support the learning of regularities embedded in the tone stream, as well as the right angular gyrus and right posterior superior temporal gyrus (STG). To our knowledge there is yet no study to investigate changes in the amount of information sharing within large-scale networks of distributed cortical regions related to statistical learning of tone sequences. Nonetheless, music training has been shown to modify the functional connectivity of cortical regions underlying the processing of other music related material^21^.

The scope of the present study was to investigate functional connectivity changes of the cortical network underlying statistical learning using MEG measurements, and to assess how this network is reorganized due to long term musical training. To this aim we re-analyzed the data of our previous study^11^ that compared statistical learning effects in musicians and non-musicians following an approach that allowed us to identify changes in the corresponding cortical network via a graph theoretical approach. Based on the fact that musicians show enhanced cortical connectivity, in comparison to non-musicians, when confronted to music-related tasks^21^, we hypothesized that under the condition of a statistical learning experiment musicians would show increased sharing of information between distributed cortical areas and hence, increased connectivity compared with the non-musicians’ network.

## Results

### Behavioral responses

The results from the behavioral responses in the statistical learning test phase are described in detail in^11^. Nonetheless, it can be mentioned that neither of the two groups performance was significantly different from the chance level [musicians: *t*(14) = −1.154, *p* > 0.05; non-musicians: *t*(14) = −1.898, *p* > 0.05; one – sample t–test].

### Graph analysis results

#### Cortical network supporting statistical learning in musicians

**Figure 1:**
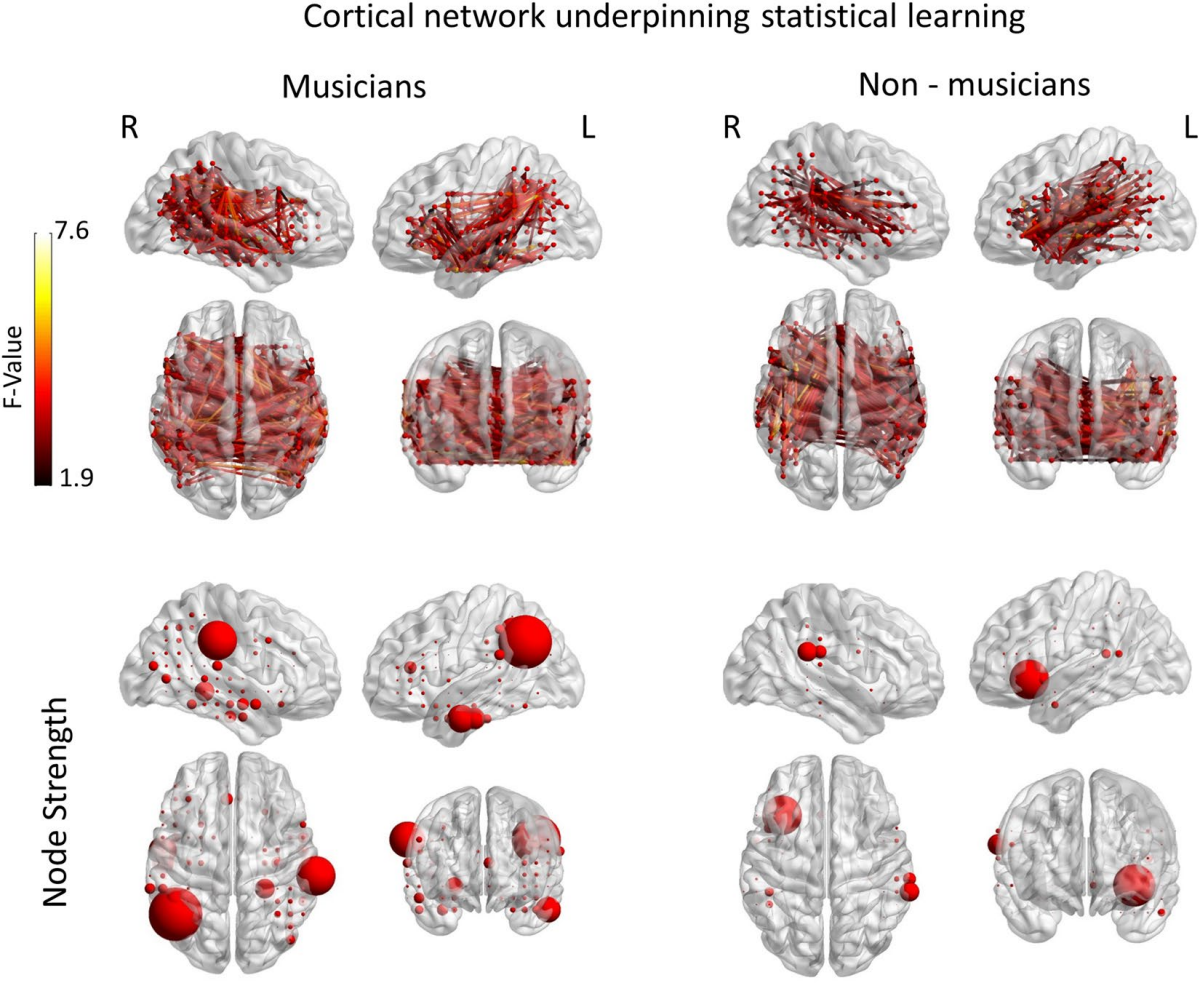
Cortical network supporting statistical learning in musicians and non-musicians. Upper side: Statistical parametric maps of the significant networks for the standard ≠ deviant comparison in musicians (left side) and non-musicians (right side). Networks presented are significant at p < 0.001, FDR corrected level. The color scale indicates f values. Lower side: Node strength of the significant networks for each comparison. Strength is represented by node size.

During the MEG measurement, two tone sequence sets were combined and randomly interleaved in an oddball paradigm: (a) the standard tone sequences which are sequences presented with a higher probability rate (probability = 0.8) and (b) the deviant ones which are sequences presented with a lower probability (probability = 0.2). The statistical analysis of the adjacency matrices of the group of musicians with the contrast of standard ≠ deviant revealed a complex network of sources [threshold: F(14) = 1.9, p < 0.001; FDR corrected]. This network consisted of 230 nodes (i.e. cortical areas which contribute in the information processing pathway of the network) out of the 266 nodes that were available in the mask, and 920 edges, which represent the connections between nodes. The network edges were mainly inter-hemispherical connecting most of the regions included in the mask (Fig. 1).

In order to investigate the qualitative parameters of the network, the graph characteristics of node strength, network density and global efficiency were calculated for the significant graphs as mentioned in the methods section. Node strength is the sum of the weights (i.e. *t* values in the present analysis) of incoming and outgoing edges connected to the node, hence, identifying the nodes with greater connectivity differences. Node density divides the present connections to all possible connections, while global efficiency is the average inverse shortest path length in the network, depicting the level of global integration in the network.

The global efficiency of the network was found to be E = 0.2129, the density of the network was found to be D = 0.0131, while the node strength identified several nodes (i.e. 12) to be of greater importance for the information flow within the network. These nodes include the posterior temporal/intraparietal lobule bilaterally, the middle temporal gyrus bilaterally, and the anterior cingulate cortex. An additional correlational analysis did not show significant correlations between the number of years of musical practice nor with age of onset of musical practice and the connectivity measures in the group of musicians.

#### Cortical network supporting statistical learning in non-musicians

The statistical analysis of the adjacency matrices of the group of non-musicians with the contrast of standard ≠ deviant revealed a network of sources [threshold: F(14) = 1.9, p < 0.001; FDR corrected] that was more confined than the musicians’ one and consisting of 808 edges and 186 nodes mainly having inter-hemispherical edges connecting all the regions included in the mask (Fig. 1). The global efficiency of the network was found to be E = 0.1428, the density of the network was found to be D = 0.0115, while the node strength identified only 5 nodes to be of greater importance for the information flow within the network. These nodes include the posterior STG bilaterally, and the left IFG.

#### Differences in the statistical learning network of musicians and non-musicians

**Figure 2:**
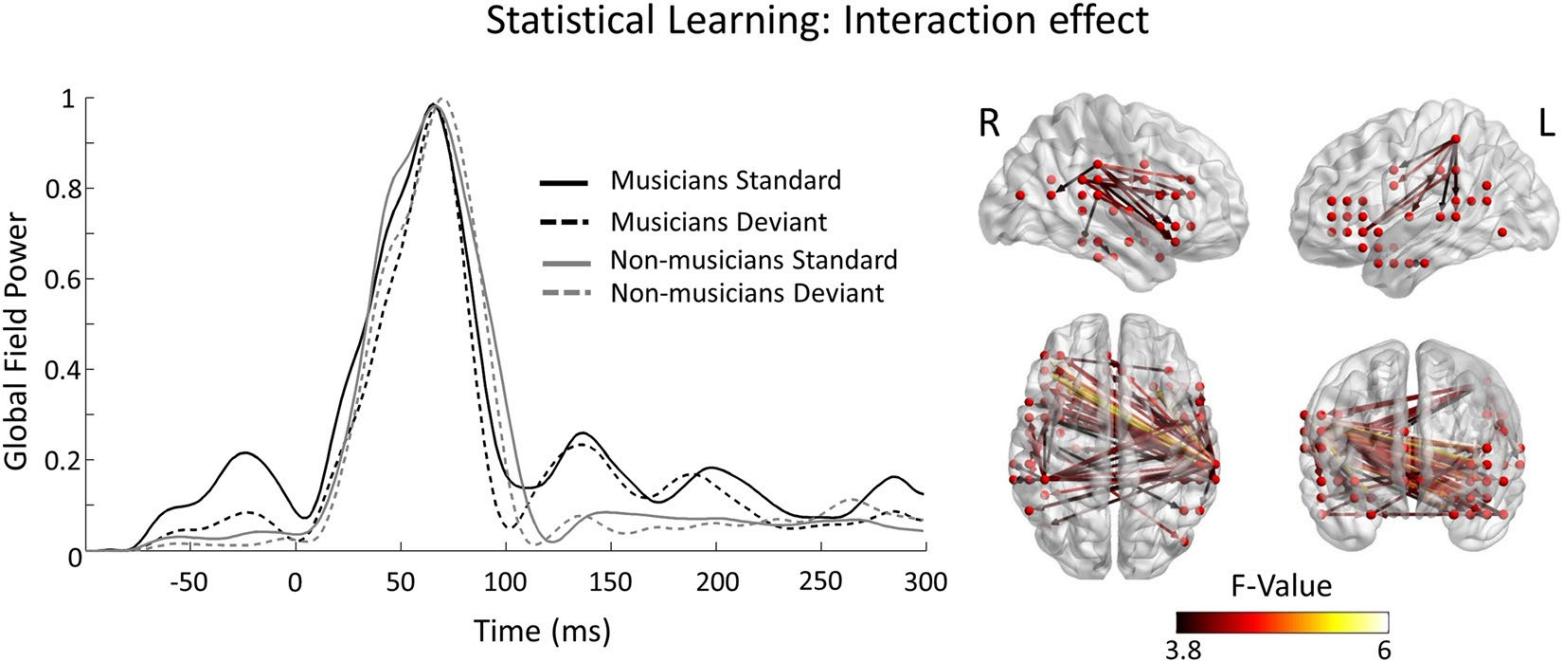
Global Field Power and group × condition interaction effect. Left side: grand average global field power for the evoked responses of musicians (continuous line) and non-musicians (dashed line) in the standard (black line) and deviant (grey line) condition. Global Field Power is depicted in order to reveal the overall sensor activation pattern. Right side: Statistical parametric maps of the network for the interaction effect of group × condition. The network presented is significant at p < 0.001, FDR corrected level. The color scale indicates f values.

The grand average of the Global Field Power (GFP) of each condition and for each group was calculated, as a gross index of the overall activity. The GFP indicated increased activity in the group of musicians in comparison to the non-musicians in the time-window of 120–220 ms (Fig. 2). The mixed model analysis of the adjacency matrices for the interaction between the factors Group (musicians and non-musicians) and Condition (standard and deviant) using the run (first, second or third) as a covariate revealed significant differences [threshold: F(1, 27) = 3.8, p < 0.001; FDR corrected] in a network of sources consisting of 59 edges and 37 nodes having both intra- and inter-hemispherical edges (Fig. 2). In both hemispheres, these edges connected the intraparietal lobule to the intra-hemispheric sources of posterior temporal and inferior frontal sources. Regarding the inter-hemispherical connections, the left posterior temporal sources were connected to the right posterior temporal/intraparietal ones, which were in turn connected to the left IFG.

**Figure 3:**
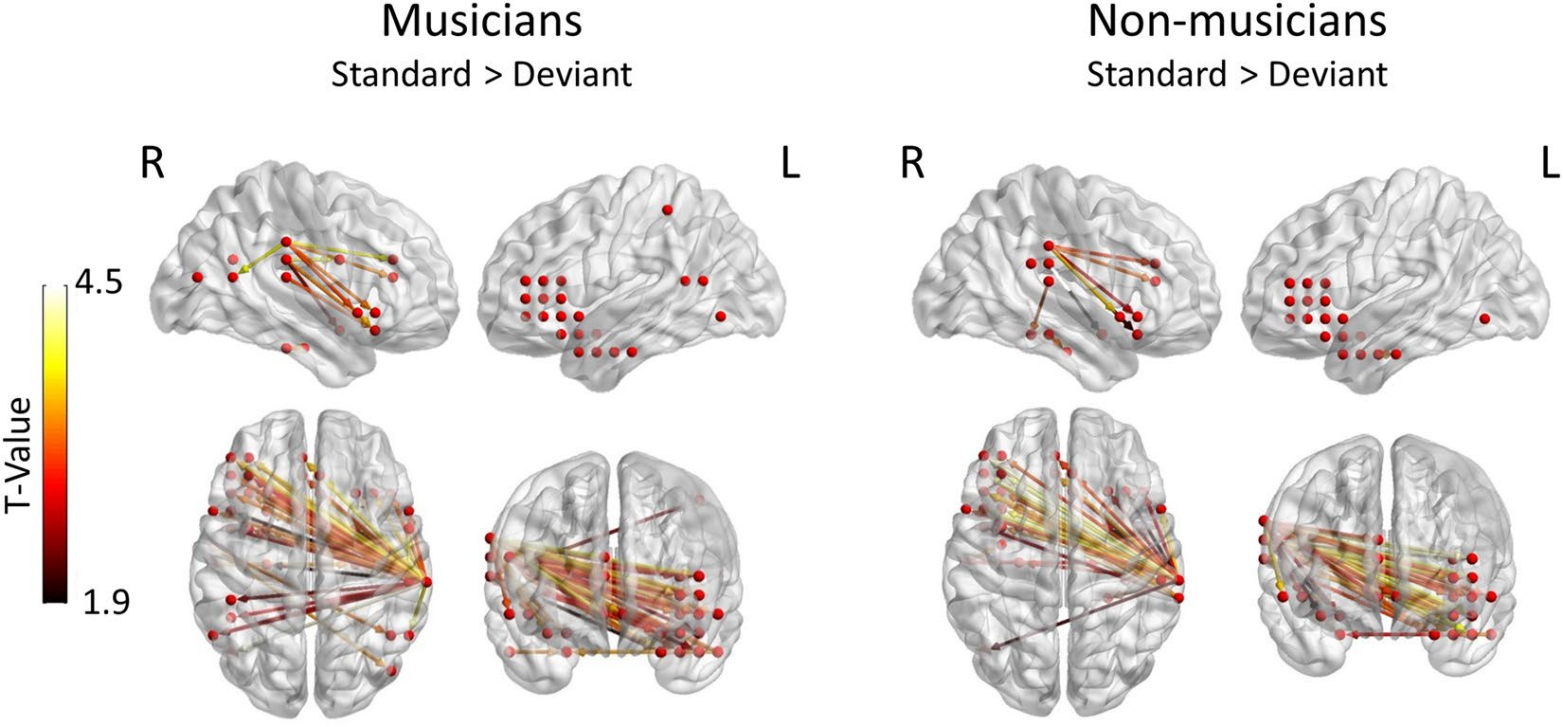
Cortical network supporting the identification of standard sequences in musicians and non-musicians. Statistical parametric maps of the significant networks for the standard > deviant comparison in musicians (left side) and non-musicians (right side). Networks presented are significant at p < 0.001, FDR corrected level. The color scale indicates t values.

To explore and understand the interaction result, we formed a mask based on the interaction effect and performed two post-hoc analyses: one comparing the different conditions within each group and one comparing the different groups within each condition. The analysis of the contrast standard > deviant within the group of musicians [threshold: t(14) = 1.9, p < 0.001, FDR corrected] mainly indicated significant connections between the left temporal, intraparietal and inferior frontal sources to the right posterior STG, and intra-hemispheric connections of right posterior temporal/intraparietal lobule to the right IFG. The analysis of the same contrast within the group of non-musicians [threshold: t(14) = 1.9, p < 0.001, FDR corrected] mainly showed significant connections between the right posterior STG and the left IFG. Intra-hemispheric connections in the right hemisphere connecting the posterior temporal/intraparietal region to the right inferior frontal region were also present (Fig. 3). The analysis of the contrast deviant > standard within each group did not yield significant connections.

**Figure 4:**
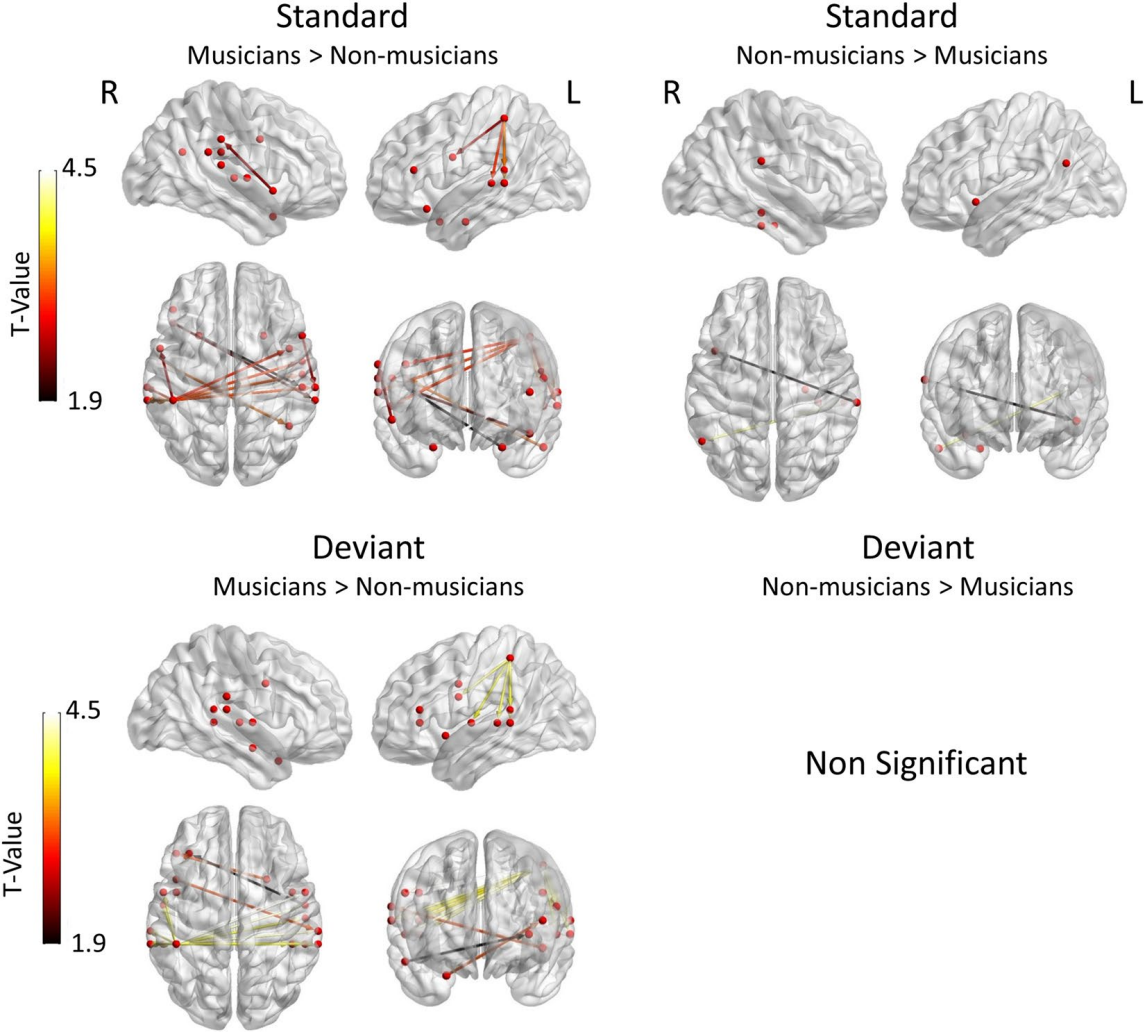
Differences in connectivity between musicians and non-musicians. Left side: Statistical parametric maps of the significant networks for the musicians > non-musicians comparison in the contrasts: standard > deviant (upper side) and deviant > standard (lower side). Right side: Statistical parametric maps of the significant networks for the non-musicians > musicians comparison in the standard > deviant (upper side) contrast. The deviant > standard contrast in this comparison did not yield significant results (lower side). Networks presented are significant at p < 0.001, FDR corrected level. The color scale indicates t values.

The analysis of the contrast musicians > non-musicians within the condition standard [threshold: t(28) = 1.9, p < 0.001, FDR corrected] revealed that musicians showed significantly enhanced connectivity in comparison to non-musicians between the left intraparietal lobule and left posterior temporal sources as well as between the left intraparietal lobule and the right STG and the left IFG. Additionally, significantly stronger connections were found in this contrast between the right STG and the left IFG. The total amount of significant edges and nodes depicting the connectivity differences in this contrast was 13 edges and 19 nodes. The analysis of the corresponding contrast of non-musicians > musicians [threshold: t(28) = 1.9, p < 0.001, FDR corrected] showed a smaller amount of connections (i.e. 3 edges and 6 nodes) mainly between the right STG and the left inferior frontal one (Fig. 4). The analysis of the contrast of musicians > non-musicians within the condition deviant [threshold: t(28) = 1.9, p < 0.001, FDR corrected] showed significant connectivity differences between the two groups in the connections of the left intraparietal lobule, the left posterior STG and the left postcentral gyrus. Inter-hemsispherically, significant connectivity differences were found between the left intraparietal lobule and the right posterior STG. Additionally, significantly greater connectivity values were found between the right STG and the left IFG (Fig. 4). The opposite contrast of non-musicians > musicians within the condition deviant yielded no significant results.

## Discussion

The present study investigated the cortical large-scale network supporting statistical learning of tone sequences. The reorganization of this network that is related to musical expertise was assessed via a cross-sectional comparison of a group of musicians to a group of non-musicians. The cortical responses to a statistical learning paradigm incorporating an oddball approach were measured via MEG recordings. Large-scale connectivity of the cortical activity was calculated via a statistical comparison of the estimated transfer entropy (TE) in the sources’ activity. A dense cortical network is shown by the statistical comparison to underpin statistical learning in non-musicians, while a different network integrating more widespread and distributed cortical areas supports the same process in musicians. Moreover, the graph organization characteristics of node strength, network density and global efficiency indicate that musicians’ network shows enhanced processing efficiency.

The behavioral results of the present study have been reported and interpreted in^11^. Nonetheless it has to be mentioned that recently in a series of studies investigating statistical learning of tone sequences^6,10,22^ the behavioral results, in contrast to the neurophysiological ones, did not reach significance and hence did not indicate that the encoding of the tone sequence sets reached the level of awareness. Further research has to be conducted in order to identify the conditions under which the result of the sequence segmentation based on transitional probabilities reaches the behavioral level. Within this framework, some researchers argue that attending to the stimulation is a necessary condition for statistical learning to be detected behaviorally^23,24^, while the structural character^6,25^, the stimulus type^22^, and the instructions provided to the subjects during the familiarization phase (incidental/intentional)^6,25,26^ seem also to be related to the behavioral outcome. In addition, it has to be noted that the task used for the assessment of learning (i.e. a 2 alternative forced choice test) may also be the cause of the absence of behavioral results, as it may not reflect the proposed process so accurately, when this is measured by deviations from the chance level^27^. Moreover, the applied procedure requires explicit decisions regarding the familiarity of each sequence without the provision of any feedback, while the presentation of non-familiar items may interfere with the implicit nature of the learning outcome^28^. Hence, implicit measures, such as the neurophysiological measurements or behavioral priming paradigms, may evaluate the learning outcome in such procedures more accurately^28^.

The cortical network analysis revealed that both groups of musicians and non-musicians engage a large-scale network consisting of a variety of distributed sources for evaluating predictable and unpredictable tone sequences on the basis of their statistical probabilities. Specifically, non-musicians revealed a network of sources having both inter- and intra-hemispherical edges. The graph analysis based on the node strength revealed that the most important nodes of the network were the posterior part of the STG bilaterally and the left IFG, identifying the importance of the role of these areas in the information processing network. This finding is consistent with the role of the primary and secondary auditory cortex in statistical learning of tone sequences as indicated by the source analysis of our previous study^11^ as well as by several other studies using source analysis of MEG^20,29^ and sensor analysis of Event Related Potentials^6,26,30,31^. Additionally to these sources, an important role for this process seems also to be served by the right temporoparietal junction^25^. The modulation of the activity of the STG is probably performed via top-down influences^32^ functioning within the framework of predictive coding^33^.

The importance of the left IFG is a finding that has been repetitively found in studies investigating statistical learning of tone sequences^16,34^. It is also consistent with the role of Broca’s area as a region processing supramodally hierarchically structured sequences^35–37^, also within the framework of music processing^38,39^. This interpretation is also in line with the view of Frost *et al*.^7^, proposing that statistical learning may be grounded both in domain-general learning principles which are constrained to operate within specific modalities based on stimulus specificity, as well as in modality-specific processing and representation areas.

Functional connectivity networks are correlated but do not necessarily coincide with the structural ones^40^. In the present results of the group of non-musicians, the intra-hemispherical connections of the left IFG originate from the posterior portion of the STG and include nodes in the middle temporal gyrus, while the inter-hemispherical ones are direct. These results may be an indication that mainly two white matter pathways are used in the information processing pattern of the cortical network supporting statistical learning of tone sequences in non-musicians: (i) the external capsule/uncinate fasciculus serving as the ventral auditory pathway^41,42^ and (ii) the corpus callosum. It has to be noted though that the right homologue of Broca’s area is known to support hierarchical structure processing^43^, a necessary process for statistical learning, and that this area is connected to the posterior portion of the STG via the arcuate fasciculus which is part of the superior longitudinal fasciculus^41^. Hence, the inter-hemispherical connections found in the present study between the right STG and the right IFG are an indication that the arcuate fasciculus may also play an important role in statistical learning. This interpretation is in line with the study by López-Barroso *et al*.^44^, which highlights the role of the left arcuate fasciculus in word learning.

The cortical network of sources that musicians activate during statistical learning indicates an extensive reorganization compared to the non-musicians one. The graph analysis shows a network that has greater density and efficiency than the network of non-musicians, while the node strength analysis revealed a greater variety of sources including the posterior temporal/intraparietal lobule bilaterally, the middle temporal gyrus bilaterally, and the anterior cingulate cortex. It has to be noted though that IFG is also an active node in the network of musicians, but it shows a reduced role in the network when compared to the non-musicians, probably due to the greater variety of sources that have a more active role in the information processing pattern. It has to be noted that the reduced node strength, and hence reduced connectivity with other nodes in the network, is not comparable with the amount of activation of this region per se, which is found in previous studies to be stronger in musicians than in non-musicians^45^. The middle temporal gyrus is also a source that has been repetitively found to correlate with statistical learning in the auditory domain^46,47^, but in relation to linguistic stimuli. To our knowledge, this is the first time that this source is shown to correlate with statistical learning of tone sequences. The intraparietal lobule has been previously correlated with statistical learning of tone sequences in the MEG study of Farthouat *et al*.^20^. The anterior cingulate cortex is associated with error detection^48^ and attentional shifts^49^ two processes that are highly related to the applied paradigm as it included an oddball condition in which detection of deviant tone sequences may have triggered the anterior cingulate response. In addition, the fact that this region seems to play a more crucial role in the network of musicians indicates that, even though they were not explicitly aware of the correct and incorrect sequences, as evidenced by the behavioral results, still treated the incorrect ones as errors.

The statistical comparison of the networks of the two groups revealed statistically significant connectivity differences in an extensive amount of edges. The post-hoc analysis indicated that these differences were mostly originating from enhanced connectivity in the group of musicians compared to the group of non-musicians. More specifically, 4 different functional connectivity networks seem to differentiate the two conditions and the two groups: 2 intra-hemispheric networks connecting the intraparietal lobule with posterior temporal sources, one inter-hemispheric connecting the right posterior STG with the left IFG and one inter-hemispheric connecting the left angular gyrus with the right STG. The right intra-hemispheric network seems to support information exchange between the connected regions when a standard tone pattern is perceived, while the left one when a deviant is perceived. The later network seems to be more active in the group of musicians. The inter-hemispheric connecting the right posterior STG with the left IFG seems to be active in both groups during the perception of tonal patterns that are learned via statistical learning. The inter-hemispheric network connecting the left angular gyrus with the right STG seems to be more active in musicians but independently of whether the tone pattern was learned via statistical learning or was a new one. These results are consistent with the proposed functionality of the corresponding regions^38,46,50^ and show that musical expertise may drive significant neuroplastic changes in the cortical networks connecting the left IFG to other nodes of the network supporting statistical learning^7^. These changes may also be the cause of the significant differences that were found in statistical learning between the two groups in the functionality of the auditory cortex in our previous study^11^.

Interestingly, the form of the identified functional networks corresponds with structural ones for which a difference between musicians and non-musicians has been previously found. Specifically both of the intra-hemispheric networks may be supported by the arcuate fasciculus, which is considered an important pathway for the processing of speech and music while it has been found to show enhanced fractional anisotropy in musicians compared to non-musicians^51^. Additionally, the inter-hemispherical differences seem to be mostly subserved by the corpus callosum and especially its middle and posterior part that has also been found to be increased in musicians compared to controls^52^.

One of the core processes underlying statistical learning is the formation of predictions based on relations between successive events^53^. According to the predictive coding framework^54^, every auditory input is compared with the currently expected one. The expectation is formed by predictions which are based on relations between successive events^53^. When a difference between the expected and the actual auditory input exists, a signal is produced that codes the prediction error. When no error occurs, there is a suppression of this signal. The results of the present study, as indicated by the significant connectivity in the standard > deviant contrast, may be interpreted as the network underlying the suppression of the prediction error signal, for auditory events. Additionally, the post-hoc comparisons of the musicians versus non-musicians networks supporting the identification of standard and deviant sequences, indicates that musical training may affect predictive coding of auditory stimuli by strengthening the connectivity of the cortical regions that produce the prediction error signal, as well as the regions that are responsible for its suppression. This is in line with a series of studies indicating that musicians show increased cortical activity to auditory mismatch responses^44,55,56^.

Moreover, recent studies indicate that corticofugal projections, which, in line with the predictive coding framework modulate the responsiveness of sub-cortical auditory regions^57^, may play an important role in statistical learning^30^. The STG and the primary auditory cortex are related to such top-down connections^58^ and the fact that they show enhanced contribution in statistical learning of tones, as indicated by the connectivity of STG as well as the P50 response presented in our previous study^11^ and in a recent study by Doikoku *et al*.^59^, may support this interpretation. The augmented response of musicians in P50 and the increased connectivity of STG with other cortical regions in the group of musicians may further indicate that long-term musical training is related to enhanced top-down shaping of low level auditory regions. It has to be noted that the current study followed a cross-sectional design to assess the long-term musical training effect, and hence cannot be conclusive on the nature versus nurture debate, regarding the origin of the group differences in the cortical connectivity. A training study with novice participants and a random group assignment would be needed to allow causal inference on the plasticity effects^60^. Additionally, the 2 sample groups of musicians and non-musicians were matched on age (Musicians: mean age = 26.93; SD = 5.87; Non-musicians: mean age = 26.47; SD = 2.53) and they were all students of the University of Muenster, ensuring a relative homogeneity of the sample, apart from the status of musical training. Nonetheless their cognitive abilities (intelligence and working memory) or socio-economic backgrounds were not explicitly tested or matched and this may also be a limitation of the current study.

## Conclusion

The present study revealed the large-scale cortical network supporting statistical learning of tone sequences additionally to the effect that musical expertise has on reorganizing this network. The brain connectivity analysis allowed the identification of the functional architecture of the network supporting the processing of statistical learning, highlighting the prominent role of informational processing pathways that connect superior temporal sources with the left IFG. Musical expertise is related to extensive reorganization of this network, as the group of musicians showed enhanced global efficiency and increased contribution of additional temporal and frontal sources in the information processing pathway.

## Methods

### Subjects

The sample of the study consisted of 30 individuals, 15 musicians and 15 non-musicians. Musicians were students of the Music Conservatory in Münster (mean age = 26.93; SD = 5.87; 4 males) having mean musical training of 16.82 years (SD = 3.87). Non-musicians had no musical training other than the school lessons (mean age = 26.47; SD = 2.53; 4 males). All subjects were right handed as tested by the Edinburgh Handedness Inventory^61^, and had normal hearing according to a clinical audiometry testing. Subjects provided written informed consent prior to their participation. The study protocol was approved by the ethics committee of the Medical Faculty of the University of Münster and the study was conducted according to the Declaration of Helsinki.

### Stimuli

Two sets of tone sequences were constructed (44.1 kHz, 16 bit) using 11 pure tones from the same octave, similarly to the tones used by Saffran *et al*.^4^. The duration of the tones was 300 ms including 10 ms rise and decay time. The first set included the following sequences: ADB, DFE, GG#A, FCF#, D#ED, CC#D. The transitional probabilities within the sequences averaged 0.64 (min = 0.25, max = 1). Across the sequences boundaries the average probability was 0.14 (min = 0.05, max 0.60). The second set included the same tones arranged so that each part-sequence was made by the first two tones of a sequence of set 1, while the last tone was new. The sequences of set 2 were: ADG#, DFF#, GG#D, FCG#, D#EG#, CC#B. The transitional probabilities within sequences of set 2 averaged 0.59 (min = 0.33; max = 1) while between the sequences 0.13 (min = 0.1; max = 0.45). The frequency range for both sequence sets was common: 261.63 Hz – 493.88 Hz. In order to avoid systematical spectral differences between the final tones of the two sets, their mean frequencies were compared, revealing no significant difference [t(10) = 0.709, p = 0.494]. Mean frequency of the final tone for set 1 was 370.136 Hz, SD = 81.89 while for the set 2 was 400.571 Hz, SD = 65.9. The frequency of occurrence of the tones in the two sets was also compared revealing no significant difference [t(5) = 0.510, p = 0.05].

## Experimental Design

The experimental design constituted a combination of a typical statistical learning and an oddball design. Specifically, an initial familiarization phase served for establishing the basic representations of the sequences that were to serve as standards. This phase lasted 1.94 min and included 180 tone sequences from one sequence set only. The ISI was set to 35 ms. This interval was also embedded between the tones of each sequence, ensuring that it cannot be used as indicator for the segmentation process. In the following, the two sequence sets were randomly interleaved in an oddball paradigm (probability = 0.2) with 2 constraints: a) at least 3 standard sequences had to occur between presentation of two deviant sequences and b) the same sequence could not occur in two successive trials. The subjects were exposed to 3 oddball runs, lasting 8.08 min each. The total number of stimuli in the oddball runs was 500 tone sequences for the standard set (including the familiarization phase) and 100 for the deviant set of sequences.

In the following, participants were informed about a surprise behavioral test phase. In this task the subjects underwent a two-alternative forced choice test in which each standard tone sequence was paired with a deviant one. The sequences in each trial were separated by 300 ms and the inter-trial interval was 3 sec. The order of presentation of the standard and deviant sequence was counterbalanced. The participants’ task was to indicate which of the two triplets was familiar to them via a button press response. The subjects were instructed to respond within 2 seconds.

### MEG recordings

Evoked magnetic fields were recorded with a 275 channel whole-head system (OMEGA, CTF Systems Inc, Port Coquitlam, Canada) in a magnetically shielded room. Data were acquired continuously during each measurement run with a sampling rate of 600 Hz and an on-line low-pass filter of 150 Hz. Subjects were seated upright, and their head position was stabilized with cotton pads inside the MEG dewar. Auditory stimulation was delivered via 60 cm long silicon tubes at 60 dB SL above the individual hearing threshold that was determined with an accuracy of 5 dB for each ear. Subjects were instructed not to pay attention to the sound stimuli and watched a soundless movie of their own choice that was projected via an Optoma EP783S DLP projector with a refresh rate of 60 Hz onto the back of a semi-transparent screen positioned approximately 90 cm in front of the subjects’ nasion. Subjects listened to the familiarization phase and the three oddball runs with short breaks in between. The continuous recording was time-locked to the presentation of the last tone of each sequence.

### MEG data analysis

Brain Electrical Source Analysis software (BESA research, version 6, Megis Software, Heidelberg, Germany) was used for the pre-processing of the MEG data. Artifacts due to blinks or eye movements were corrected by applying an adaptive artifact-correction^62^. The continuous data were separated in epochs of 400 ms, starting 100 ms before the critical tone (last tone of each sequence) and ending 300 ms after the tone onset. Data were filtered offline with a high pass forward filter of 1 Hz and a low pass zero-phase of 30 Hz. All epochs were baseline adjusted based on the 35 ms before tone onset, as the time-interval of −100 to −35 ms included the response on the previous tone. Epochs containing signals larger than 2.5 pT were considered as artifacts and were excluded from the averaging. The subset of standards directly preceding the deviants was used in the averaging of the standards so that the two conditions (standards and deviants) share a similar signal to noise ratio.

### Source activity estimation

Current density reconstructions (CDRs) were calculated on the neural responses of each subject separately for each run’s standard and deviant averages using LORETA method^63^ as provided by BESA. LORETA directly computes a current distribution throughout the full brain volume. This method has been used successfully for the mapping of auditory oddball paradigms^64–66^ and has the advantage of not needing an a priori definition of the number of activated sources. Its individual’s CDRs were calculated for the complete response time-window (i.e. 0–300 ms) of each condition, each run and for each sample point using an average, finite element head model. It has to be noted that the accuracy of source analysis in MEG data is less susceptible to forward modeling errors, than EEG data, when a realistic head model is used^67^. The CDRs were exported for each subject and each condition (standard and deviant).

On these images a mask has been applied including the STG, inferior parietal lobule, IFG, inferior temporal gyrus, parahippocampal gyrus, middle temporal gyrus and anterior cingulate. This was performed, in order to achieve a balance between (a) restricting the solution to a physiologically plausible space, (b) reducing the final amount of nodes included in the network, and at the same time (c) allowing the LORETA solution to identify significantly activated sources. The choice of the regions that were included in the mask was based on prior MEG^20^ and fMRI localization studies^7,15,46,47^ which focused on the amount of activation of the brain areas involved in statistical learning. This procedure resulted in a source space of 266 voxels.

### Graph analysis

The CDR voxel time-series were extracted in order for the connectivity matrices to be calculated, while a node of the network was appointed to each voxel. The Matlab ® R2010a (The MathWorks, Inc., Massachusetts, United States.) toolbox HERMES^68^ was used for calculating the 266 × 266 adjacency matrix from the voxel time-series of each subject and each condition based on the algorithm of Transfer Entropy (TE). ΤΕ is a non-parametric statistic measuring the amount of directed (time-asymmetric) transfer of information between two random processes. The main advantage of TE is that, being based on probability distributions, it detects higher order correlations. Therefore, its result is not dependent on any specific model of the data^68,69^.

The Network Based Statistic (NBS)^70^ toolbox was used to identify statistically significant connections in the networks. Specifically, a 2 × 2 mixed model ANOVA with between subject factor group (Musicians and non-musicians) and within subject factor condition (Standard and deviant), using run as a covariate (oddball run 1, oddball run 2 and oddball run 3), was designed to explore the main effect of condition within each group and the group × condition interaction. The significance level was set to *p* < 0.001 corrected for multiple comparisons via FDR correction. The graph characteristics of node strength, network density and global efficiency were calculated using the Brain Connectivity Toolbox^71^. The visualization of the significant networks as directed and weighted graphs was done using BrainNet Viewer^72^.

### Data Availability

The datasets generated during and/or analysed during the current study are available from the corresponding author on reasonable request.
